# Correction to ‘Securing a furan based biorefinery: Disclosing the genetic basis of the degradation of hydroxymethylfurfural and its derivatives in the model fungus *Aspergillus nidulans*’

**DOI:** 10.1111/1751-7915.14391

**Published:** 2024-01-18

Celso Martins, Diego O. Hartmann, Adélia Varela, Jaime A. S. Coelho, Pedro Lamosa, Carlos A. M. Afonso, Cristina Silva Pereira. *Microbial Biotechnology*, 13, 1689–2078. Doi: 10.1111/1751‐7915.13649

Acknowledgements:

This work was financially supported by the projects: LISBOA‐01‐0145‐FEDER‐007660 (Microbiologia Molecular, Estrutural e Celular) funded by FEDER funds through COMPETE2020 – Programa Operacional Competitividade e Internacionalização (POCI) and by national funds through FCT (Fundação para a Ciência e a Tecnologia), PTDC/AGR‐TEC/1191/2014, funded by FCT and ‘PinusResina’ no. PDR2020‐101‐031905, funded by PDR2020 through Portugal2020. CM, DOH and JASC are grateful for the fellowships SFRH/BD/118377/2016, SFRH/BPD/121354/2016 and SFRH/BPD/100433/2014 and from European Research Area Net‐work; ERANet LAC (ref. ELAC2014/BEE‐0341). The NMR data were acquired at CERMAX, ITQB NOVA, Oeiras, Portugal with equipment funded by FCT. The authors are extremely thankful to Maria C. Leitão (ITQB NOVA) for meaningful support in chromatographic analyses.

In paragraph 4 of the ‘Acknowledgements’ section, the text ‘PTDC/AGR‐TEC/1191/2014’ is incorrect. This should have read: ‘DeepBiorefinery (PTDC/AGR‐TEC/1191/2014) funded by FEDER through “Programa Operacional Competitividade e Internacionalização” and by National Funds through FCT‐Fundação para a Ciência e a Tecnologia within the Project POCI‐01‐0145‐FEDER‐16549,’

We apologize for this error.

